# Poor mental health days and depressive disorders comparing non-caregivers, informal non-cancer caregivers, and informal caregivers of cancer patients: BRFSS 2022–23

**DOI:** 10.1007/s00520-026-11337-y

**Published:** 2026-10-14

**Authors:** Jeanne M. Ward, John R. Blosnich, Victoria Champion

**Affiliations:** 1 Indiana University School of Nursing, 600 Barnhill Drive, Indianapolis, IN 46202 USA; 2 University of Southern California Suzanne Dworak-Peck School of Social Work, Los Angeles, CA USA

**Keywords:** Mental health, Caregiving, Cancer care, Health promotion, Public health, BRFSS

## Abstract

**Purpose:**

Informal caregivers are increasing in number, especially those caring for cancer patients, and caregiving can affect both patient outcomes and caregiver well-being. This study sought to elucidate whether the mental health outcomes differed among informal cancer caregivers, informal non-cancer caregivers, and non-caregivers.

**Methods:**

Data from 2022 and 2023 Behavioral Risk Factor Surveillance Surveys (BRFSS) were analyzed for 24 states administering the caregiving module. The sample included non-caregivers(*n* = 157,539), informal non-cancer caregivers (*n* = 39,047), and informal cancer caregivers (*n* = 3,078). Logistic regressions identified factors associated with frequent mental distress days (FMD; ≥ 6 days of poor mental health in the last 30 days) and lifetime depression diagnosis. Models included caregiver status, reporting ≥ 14 poor physical health days monthly, exercise in the previous month, age, sex, marital status, education, employment, and rural/urban status, adjusted for U.S. region and year.

**Results:**

In unadjusted comparisons, informal caregivers differed significantly from non-caregivers across several sociodemographic and health characteristics and were more likely to report FMD or a lifetime depression diagnosis. After adjusting for sociodemographics, year, U.S. region, physical health, and exercise, informal cancer caregivers had significantly higher odds of FMD than non-caregivers (aOR = 1.89, 95%CI = 1.58–2.26). Informal non-cancer caregivers had significantly higher odds of a lifetime depression diagnosis (aOR = 1.44, 95%CI = 1.36–1.52) than non-caregivers.

**Conclusion:**

Cancer caregiving was more strongly associated with frequent mental distress than with lifetime depression. Integrating clinical screening into patient visits and informal caregiver supports could help identify caregiving distress during periods of increased burden and facilitate timely supportive care.

**Supplementary Information:**

The online version contains supplementary material available at https://doi.org/10.1007/s00520-026-11337-y.

## Introduction

Informal caregivers consist of family members, friends, or other individuals who support patients’ complex needs outside the formal healthcare system, [1, 2] and they play a critical role in supporting cancer patients. There are approximately 2.8 million informal caregivers of cancer patients [3], and that number is expected to grow alongside the more than two million new cancer diagnoses in 2026 [4, 5]. Informal caregivers substantially contribute to cancer patients’ outcomes, including improving patients’ mental health and coping with the adverse effects of treatment [1, 6]. However, many of these caregivers’ psychological needs are unmet, resulting in poor mental health [7, 8]. Informal cancer caregivers also carry a significant burden due to the demands of caregiving, psychologically, financially, and physically [2]. Emerging data show that informal caregivers of cancer patients have mental health needs that exceed those of the general population [9, 10], but population-level comparisons remain limited. Additionally, more recent estimates are needed, especially after the height of the COVID-19 pandemic.

Multiple factors may be associated with mental health outcomes, including physical health status, exercise, emotional support, employment status, and other socio-demographic characteristics, such as age, sex, income, education level, and marital status [11]. Many informal caregivers report feeling emotional distress due to their grief and other factors related to the patient situation, such as upsetting information from clinicians or a rapid decline in patient status [10]. Other studies about informal cancer caregivers cite unmet needs for financial, psychosocial, community, or religious support [12, 13], which can also contribute to mental distress. In turn, frequent mental distress (FMD), defined as having six or more poor mental health days in the last 30 days [14–17], is associated with multiple adverse health behaviors, increased healthcare utilization, mental health disorders, and chronic disease [17]. Other studies of informal cancer caregivers’ mental health have not compared their outcomes to those of the general population [8, 18, 19]. Despite the importance of these other outcomes, it remains unclear how the mental health of informal cancer caregivers differs from that of the general population, and what salient factors are related to self-disclosed mental distress and depression diagnoses. These group comparisons are important for public health benchmarking to detect potential disparities and identify areas of intervention.

The health and well-being of informal cancer patient caregivers are commonly conceptualized through multiple outcomes, including depression and emotional distress [20]. The Cancer Family Caregiving experience model provides a conceptual framework to understand cancer patient caregivers' stressors and well-being outcomes (mental health days or depression) [20]. In this framework, contextual factors (socio-demographics) and primary and secondary stressors culminate in caregivers' cognitive and behavioral responses within the stress process. Primary stressors include increased cancer caregiving demands, such as psychomotor tasks and driving to more appointments as patient acuity increases, and secondary stressors include spillover onto employment and finances. This process occurs over the trajectory of the cancer patient’s illness process, affecting the health and well-being of the caregiver, including their mental health. While this theoretical underpinning is important, population-level exploration remains limited to operationalizing these factors and testing their association with outcomes.

The purpose of this study was to understand the factors associated with reporting frequent mental distress or a depressive disorder diagnosis and whether these differ between non-caregivers, non-cancer informal caregivers, and informal cancer caregivers, utilizing a national dataset. We hypothesized that all informal caregivers would have higher odds of poor mental health outcomes than the non-caregiving population, and that informal caregivers of cancer patients may differ from other informal caregivers. This information will provide oncology and mental health providers with data on factors most strongly associated with poor mental health outcomes of informal caregivers. This analysis will also inform the development of screenings and interventions for informal cancer caregivers.

## Methods

### Design and sample

The Behavioral Risk Factor Surveillance Survey (BRFSS) is an annual health behavior survey administered by states through coordination by the Centers for Disease Control and Prevention (CDC). The survey instrument consists of core items that states must administer, and states can use optional modules. In 2022 and 2023, 24 states or territories collected information using the CDC’s optional caregiving module. In 2022, Puerto Rico and the following states used the module: Arizona, Colorado, Georgia, Louisiana, Mississippi, New Hampshire, Ohio, Oklahoma, Oregon, Pennsylvania, Utah, Virginia, Washington, and Wisconsin. In 2023, the following states used the module: Arizona, Arkansas, Hawaii, Idaho, Iowa, Louisiana, Maine, Maryland, New York, Ohio, Oregon, Tennessee, and Texas. Individuals from states administering survey versions with the caregiving modules and who described their caregiving status (*n* = 199,664) met the inclusion criteria for this study. The combined response rates for the 24 states across both years ranged from a low of 31% in Tennessee in 2023 to a high of 58.5% in Puerto Rico in 2022. Further details about the BRFSS methodology are available from the CDC [21].

### Independent variables

Demographic variables for all analyses included age, sex, race/ethnicity, marital status, educational level, and urban/rural status. Age categories were 18–24 years, 25–34 years, 35–44 years, 45–54 years, 55–64 years, and 65 years or older. Race included categories of non-Hispanic White, non-Hispanic Black, non-Hispanic American Indian/Alaska Native, Hispanic, non-Hispanic Multiracial or Other, and non-Hispanic Asian/Pacific Islander/Native Hawaiian. Marital status was categorized as married or partnered, divorced or separated, widowed, or never married. Education level was defined as less than high school, high school graduate, some college, or a college or technical school graduate. Employment status was recoded as employed (including self-employed), unemployed, or out of the workforce (i.e., homemakers, students, retirees, and individuals unable to work). Geography was defined as urban or rural, using the National Center for Health Statistics (NCHS) Urban–Rural Classification, considering large central, large fringe, medium, and small metropolitan areas in addition to micropolitan areas as urban, and noncore non-metropolitan areas as rural [22]. To account for regional differences in caregiving support, states were coded into the following regions: West (Arizona, Utah, Colorado, Texas, Oregon, Washington, Hawaii, and Idaho), Midwest (Ohio, Wisconsin, Oklahoma, and Iowa), South (Louisiana, Georgia, Mississippi, Virginia, Arkansas, Maryland, Tennessee, and Puerto Rico), and Northeast (New Hampshire, Pennsylvania, Maine, and New York). Year was separated into survey years of 2022 and 2023.

Independent variables also included selected health behaviors. Exercise in the previous month was assessed with the following yes/no question: “During the past month, other than your regular job, did you participate in any physical activities or exercises such as running, calisthenics, golf, gardening, or walking for exercise?” The BRFSS questionnaire defined poor physical health as physical illness or injury, asking respondents: “Now thinking about your physical health, which includes physical illness and injury, for how many days during the past 30 days was your physical health not good?” Poor physical health days were dichotomized into reporting 14 or more poor physical health days per month or less than 14 poor physical health days per month [23].

The main independent variable was informal caregiving status, which was assessed with a yes/no question: “During the past 30 days, did you provide regular care or assistance to a friend or family member who has a health problem or disability?” Individuals who answered “Yes” proceeded with the caregiving module of the BRFSS. Only versions of the BRFSS surveys used by states assessing the caregiver status question were included in the analysis, listed in Supplementary Table 1.

The caregiving module asked caregivers to select one response to the following question: “What is the main health problem, long-term illness, or disability that the person you care for has?” Caregivers could select from 14 options: arthritis or rheumatism, asthma, cancer, chronic respiratory conditions (e.g., emphysema, chronic obstructive pulmonary disorder), cognitive disorders (e.g., Alzheimer’s disease, dementia), developmental disabilities (e.g., autism, Down’s syndrome, spina bifida), diabetes, vascular issues (heart disease, hypertension, and stroke), Human Immunodeficiency Virus (HIV), mental illnesses (anxiety, depression, and schizophrenia), organ failure or diseases, substance abuse or addiction disorders, injuries, and old age or infirmity. We separated individuals who identified as cancer caregivers from non-cancer caregivers and compared them with non-caregiving individuals.

### Dependent variables

The BRFSS assessed mental health with the following question: “Now, thinking about your mental health, which includes stress, depression, and problems and emotions, for how many days during the past 30 days was your mental health not good?” Poor mental health days were then operationalized as six or more days of poor mental health per month, indicating frequent mental distress (FMD6) or fewer than six days of poor mental health per month. The FMD6 (FMD) indicator has been validated against standards of mental distress (i.e., the Kessler 6 scale of mental distress) and effectively used with multiple populations [15, 16, 24]. The second dependent variable was whether an individual had a lifetime depression diagnosis (yes/no), assessed by the BRFSS with the following question: “(Ever told) (you had) a depressive disorder (including depression, major depression, dysthymia, or minor depression)?

### Data analysis

The datasets from the BRFSS were downloaded and combined into one file, reflecting the appropriate weights for each survey version. Arizona and Ohio utilized more than one survey version, so a working dataset was created following the BRFSS complex sampling recommendations for survey weights [25]. We created a weight field containing 2022 or 2023 weights for each state’s data, and all analyses were weighted. Missing data were retained by being recoded into other/unknown categories for each variable. We used Pearson’s chi-squared tests to compare cancer caregivers with the general population on the prevalence of each socio-demographic variable, exercise, physical health indicator, and mental health outcomes (i.e., FMD and depression), and calculated descriptive effect sizes with Cramer’s V using the uncorrected Pearson chi-square. Logistic regression was used to determine the adjusted odds of FMD or lifetime depression diagnosis by caregiver status, adjusting all models for socio-demographic covariates, physical health indicators, and exercise. Adjusted odds ratios (aOR) and 95% confidence intervals are reported.

Stata version 18 was used for all data cleaning, coding, and analysis. Statistical significance was assessed at *p* < 0.05. Because this study used a publicly available dataset, the IRB of [institution name masked] deemed the project as not human subjects research.

## Results

After identifying states using the caregiving modules, 199,664 individuals were included in unadjusted analyses, including 156,539 non-caregivers, 39,047 informal non-cancer caregivers, and 3,078 informal cancer caregivers. For adjusted analyses, the sample sizes were 186,767 and 188,345 (FMD and depression, respectively). The informal caregivers’ total numbers from each state (Supplementary Table 1) were checked for accuracy against original year datasets and available BRFSS data from individual states, including Arizona [26, 27], New York [28], Washington (Crawbuck, G., personal communication, June 16, 2025), and Wisconsin [29]. Unadjusted analyses identified differences between cancer caregivers and the general population in the prevalence of each variable. In unadjusted models, cancer caregivers were significantly more likely than the non-caregivers and non-cancer caregivers to be female (*F* = 131.83, *p* < 0.001, Cramér’s *V* = 0.13), White non-Hispanic (*F* = 22.27, *p* < 0.001, Cramér’s *V* = 0.15), 65 years or older (*F* = 42.60, *p* < 0.001, Cramér’s *V* = 0.17), married or partnered (*F* = 19.78, *p* < 0.001, Cramér’s *V* = 0.10), have attended or graduated from college or technical school (*F* = 15.76, *p* < 0.001, Cramér’s *V* = 0.09), be unemployed or out of the workforce (*F* = 9.88, *p* < 0.001, Cramér’s *V* = 0.06), live in a rural area (*F* = 5.68, *p* = 0.005, Cramér’s *V* = 0.03), and report FMD (*F* = 118.18, *p* < 0.001, Cramér’s *V* = 0.12). Non-cancer caregivers of were significantly more likely than non-caregivers and cancer caregivers to have 14 or more poor physical health days per month (*F* = 38.46, *p* < 0.001, Cramér’s *V* = 0.07) or a lifetime depression diagnosis (*F* = 120.31, *p* < 0.001, Cramér’s *V* = 0.12) (Table 1). The ranges of Cramér’s *V* varied by factor. Several were < 0.06, indicating no or very weak associations, or from 0.06 to 0.10, indicating weak associations. Values of 0.11 to 0.15 had moderate associations, and one factor (age group) had a strong effect size interpretation with a value of 0.17. [30]

**Table 1:** Prevalence in socio-demographics, frequent mental distress, and a lifetime depression diagnosis, non-caregiving population versus non-cancer informal caregivers (ICs) vs. informal cancer caregivers, BRFSS 2022–2023

	Full population *N* = 199,664		Noncaregiving population *N* = 157,539		Non-Cancer (ICs) *N* = 39,047		Cancer ICs *N* = 3,078		
*Socio-demographics*	*n*	(%)	*n*	(%)	*n*	(%)	*n*	(%)	*P*
Gender									<.001
Men	92,515	(48.4)	75,929	(50.4)	15,464	(41.1)	1,122	(39.0)	
Women	107,149	(51.6)	81,610	(49.6)	23,583	(58.9)	1,956	(61.0)	
Race/ethnicity									<.001
White (non-Hispanic)	146,260	(60.1)	114,467	(59.1)	29,421	(63.8)	2,372	(64.5)	
Black (non-Hispanic)	13,670	(11.5)	10,475	(11.1)	2,957	(13.1)	238	(14.1)	
AI/AN (non-Hispanic)	2,352	(1.2)	1,769	(1.1)	548	(1.5)	35	(0.6)	
Asian (non-Hispanic)	6,520	(4.8)	5,544	(5.3)	924	(2.9)	52	(2.9)	
Multiracial/Other (non-Hispanic)	5,830	(3.5)	4,391	(3.3)	1,327	(4.1)	112	(4.5)	
Hispanic	20,246	(16.7)	17,187	(17.9)	2,852	(12.1)	207	(11.4)	
Unknown	4,786	(2.3)	3,706	(2.3)	1,081	(2.5)	62	(1.9)	
Age group									<.001
18–24 years	11,085	(11.6)	9,474	(12.6)	1,497	(8.1)	114	(7.0)	
25–34 years	20,132	(16.1)	17,089	(17.0)	2,844	(12.8)	199	(9.9)	
35–44 years	25,812	(16.3)	20,760	(16.5)	4,686	(15.4)	366	(14.4)	
45–54 years	29,679	(15.3)	22,717	(14.8)	6,470	(17.3)	492	(16.9)	
55–64 years	36,746	(16.4)	27,273	(15.2)	8,811	(21.1)	662	(20.8)	
65 + years	76,210	(24.3)	60,226	(23.9)	14,739	(25.3)	1,245	(31.0)	
Marital status									<.001
Married/partnered	111,610	(55.2)	85,677	(54.2)	24,041	(59.3)	1,892	(59.6)	
Divorced/sep	30,469	(12.9)	23,933	(12.7)	6,063	(14.0)	473	(14.9)	
Widowed	22,475	(7.3)	19,011	(7.8)	3,154	(5.6)	310	(6.9)	
Never married	33,480	(23.6)	27,595	(24.5)	5,504	(20.3)	381	(17.9)	
Unknown	1,630	(0.9)	1,323	(0.9)	285	(0.8)	22	(0.7)	
Education level (highest)									<.001
< High school	11,215	(11.0)	9,398	(11.7)	1,678	(8.4)	139	(7.9)	
High school graduate	49,426	(27.9)	39,793	(28.2)	8,898	(27.0)	735	(25.5)	
Some college/tech school	56,056	(30.4)	43,119	(29.6)	12,043	(33.8)	894	(33.5)	
Graduate coll./tech school	82,230	(30.2)	64,607	(30.1)	16,321	(30.4)	1,302	(33.0)	
Unknown	737	(0.4)	622	(0.4)	107	(0.4)	8	(0.1)	
Employment status									<.001
Employed	97,954	(56.2)	78,041	(57.0)	18,487	(53.5)	1,426	(49.1)	
Unemployed	7,376	(5.0)	5,522	(4.8)	1,742	(5.6)	112	(6.2)	
Out of workforce	92,668	(37.6)	72,619	(36.9)	18,524	(39.9)	1,525	(44.2)	
Unknown	1,666	(1.2)	1,357	(1.2)	294	(1.0)	15	(0.5)	
Exercise in previous month									<.001
Yes	150,639	(75.1)	117,977	(74.6)	30,254	(77.2)	2,408	(77.1)	
No	48,588	(24.6)	39,206	(25.2)	8,717	(22.7)	665	(22.7)	
Unknown	437	(0.2)	356	(0.3)	76	(0.1)	5	(0.2)	
Poor physical health days									<.001
Less than 14/month	166,589	(86.7)	132,093	(87.4)	31,944	(83.9)	2,552	(84.3)	
14 or more/month	28,129	(13.3)	21,404	(12.6)	6,249	(16.1)	476	(15.7)	
Geography									.005
Urban	174,079	(93.4)	137,191	(93.6)	34,224	(92.9)	2,664	(91.2)	
Rural	20,228	(6.6)	15,610	(6.4)	4,255	(7.1)	363	(8.8)	
Year of survey									<.001
2022	116,291	(44.9)	92,627	(45.5)	21,903	(42.7)	1,761	(43.2)	
2023	83,373	(55.1)	64,912	(54.5)	17,144	(57.3)	1,317	(56.8)	
Region of U.S									
West	83,946	(35.6)	66,511	(35.9)	16,221	(34.7)	1,214	(34.5)	<.001
Midwest	48,827	(19.6)	38,674	(19.6)	9,421	(20.1)	732	(16.5)	
South	47,063	(25.7)	36,585	(25.2)	9,664	(27.2)	814	(29.3)	
Northeast	19,828	(19.1)	15,769	(19.3)	3,741	(18.0)	318	(19.6)	
Frequent Mental Distress									<.001
Less than 6/month	154,035	(76.1)	124,022	(77.6)	27,897	(70.1)	2,116	(68.8)	
6 or more/month	41,703	(23.9)	30,360	(22.4)	10,446	(29.9)	897	(31.2)	
Lifetime Depression Diagnosis									<.001
Yes	44,054	(22.1)	32,441	(20.6)	10,769	(27.9)	844	(26.0)	
No	154,448	(77.9)	124,208	(79.4)	28,026	(72.1)	2,214	(74.0)	

AI/AN = American Indian & Alaska Native. West U.S. = AZ, UT, CO, TX, OR, WA, HI, and ID; Midwest U.S. = OH, WI, OK, IA; South U.S. = LA, GA, MS, VA, AK, MD, TN, PR; Northeast U.S. = NH, PA, ME, NY

Results of regression analysis identified the association between caregiver status with mental health dependent variables while controlling for physical health, exercise, and socio-demographics. The full models were statistically significant (*p* < 0.001), indicating that they could distinguish between reporting FMD and not reporting it, and between reporting a lifetime depression diagnosis and not reporting it. Table 2 shows the significant predictors in the logistic models for the FMD and lifetime depression diagnosis outcome variables. Informal caregivers were significantly more likely than non-caregivers to have FMD, with cancer caregivers having 89% greater odds and non-cancer caregivers 58% having greater odds. Informal caregivers were significantly more likely than non-caregivers to have a lifetime depression diagnosis, with cancer caregivers having 34% greater odds and non-cancer caregivers having 44% greater odds.

**Table 2:** Associations of caregiver status and sociodemographics with FMD and lifetime depression diagnosis

	FMD6		Diagnosed with depression	
	(*n* = 186,767)		(*n* = 188,345)	
	aOR	(95%CI)	aOR	(95%CI)
Status				
Non-caregivers	Ref		Ref	
Non-cancer caregivers	1.58	(1.48–1.67)*	1.44	(1.36–1.52)*
Cancer patient caregivers	1.89	(1.58–2.26)*	1.34	(1.12–1.60)*
Age group				
18–24 years	Ref		Ref	
25–34 years	0.86	(0.78–0.94)*	1.09	(0.99–1.21)
35–44 years	0.59	(0.53–0.65)*	0.86	(0.77–0.95)*
45–54 years	0.39	(0.35–0.43)*	0.65	(0.58–0.73)*
55–64 years	0.26	(0.24–0.29)*	0.50	(0.45–0.56)*
65 + years	0.15	(0.13–0.16)*	0.28	(0.25–0.32)*
Sex				
Male	Ref		Ref	
Female	1.60	(1.52–1.69)*	2.05	(1.95–2.16)*
Race				
White (Non-Hispanic)	Ref		Ref	
Black (Non-Hispanic)	0.80	(0.74–0.87)*	0.49	(0.44–0.53)*
AI/AN (Non-Hispanic)	1.00	(0.82–1.21)	0.83	(0.68–1.01)
Asian (Non-Hispanic)	0.66	(0.57–0.77)*	0.36	(0.30–0.42)*
Multiracial/Other (Non-Hispanic)	1.15	(1.01–1.31)*	1.04	(0.92–1.18)
Hispanic	0.74	(0.67–0.81)*	0.57	(0.52–0.64)*
Unknown	0.98	(0.83–1.17)	0.66	(0.56–0.77)*
Marital status				
Married/partnered	Ref		Ref	
Divorced/separated	1.67	(1.55–1.79)*	1.84	(1.71–1.97)*
Widowed	1.38	(1.25–1.54)*	1.30	(1.18–1.45)*
Never married	1.55	(1.45–1.66)*	1.41	(1.31–1.51)*
Unknown	1.23	(0.94–1.60)	1.15	(0.87–1.52)
Education level				
< High school	Ref		Ref	
High school graduate	0.93	(0.83–1.04)	0.88	(0.78–0.99)*
Some college/tech. school	1.03	(0.92–1.16)	1.05	(0.93–1.18)
Graduate college/tech. school	0.84	(0.75–0.94)*	0.91	(0.80–1.03)
Unknown	1.08	(0.66–1.75)	0.48	(0.32–0.72)*
Employment status				
Employed	Ref		Ref	
Unemployed	1.59	(1.42–1.77)*	1.76	(1.57–1.97)*
Out of workforce	1.22	(1.14–1.30)*	1.49	(1.40–1.59)*
Unknown	0.78	(0.62–1.00)*	0.76	(0.55–1.06)
Geography				
Urban	Ref		Ref	
Rural	0.83	(0.76–0.91)*	0.87	(0.80–0.95)*
Exercise in previous month				
Yes	Ref		Ref	
No	1.25	(1.18–1.34)*	1.30	(1.22–1.38)*
Unknown	1.07	(0.63–1.81)	1.24	(0.79–1.95)
Poor physical health days				
Less than 14 days/month	Ref		Ref	
14 or more days/month	4.70	(4.38–5.03)*	2.72	(2.54–2.91)*
U.S. Region				
West	Ref		Ref	
Midwest	0.98	(0.93–1.04)	1.06	(1.00–1.12)
South	1.00	(0.94–1.06)	1.03	(0.97–1.08)
Northeast	0.84	(0.77–0.92)*	0.83	(0.76–0.91)*
Year				
2022	Ref		Ref	
2023	0.96	(0.92–1.01)	0.94	(0.90–0.99)*

AI/AN = American Indian & Alaska Native. West U.S. = AZ, UT, CO, TX, OR, WA, HI, and ID; Midwest U.S. = OH, WI, OK, IA; South U.S. = LA, GA, MS, VA, AK, MD, TN, PR; Northeast U.S. = NH, PA, ME, NY

^*^ *p* <.05

Other significant correlates included lack of exercise in the last month being associated with higher odds of FMD (aOR = 1.25; 95%CI = 1.18–1.34) and lifetime depression diagnosis (aOR = 1.30; 95%CI = 1.22–1.38), and 14 or more poor physical health days in the last 30 days being significantly positively associated with both FMD (aOR = 4.70; 95%CI = 4.38–5.03) and a lifetime depression diagnosis (aOR = 2.72; 95%CI = 2.54–2.91). Individuals from rural areas had significantly lower odds of FMD (aOR = 0.83; 95%CI = 0.77–0.91) and a lifetime depression diagnosis (aOR = 0.87; 95%CI = 0.80–0.95) than individuals from urban areas. Additionally, individuals from the Northeast region of the U.S. had significantly lower odds of FMD (aOR = 0.84; 95%CI = 0.77–0.92) and a lifetime depression diagnosis (aOR = 0.83; 95%CI = 0.76–0.91) than those from the Western region. Women had significantly higher odds of FMD (aOR = 1.60; 95%CI = 1.52–1.69) or a lifetime depression diagnosis (aOR = 2.05; 95%CI = 1.95–2.16).

## Discussion

This population-based analysis of BRFSS data from 24 states examined whether informal cancer and non-cancer caregivers have poorer mental health outcomes than non-caregivers, after adjusting for covariates. Cancer and non-cancer informal caregivers had several significant unadjusted differences with the non-caregivers, and they had significantly higher odds of FMD and a lifetime depression diagnosis than the non-caregivers in the adjusted models, underscoring the persistent burden associated with caregiving.

The stronger association observed for informal cancer caregivers having higher odds of FMD versus lifetime depression diagnosis is notable, especially given that FMD reflects distress in the last 30 days, and lifetime depression diagnosis captures a historical clinical diagnosis. This may reflect ongoing situational stress related to cancer caregiving burden rather than pre-existing depression, or it could reflect underdiagnosis of depression among cancer caregivers or barriers to accessing mental healthcare. The results here aligned with the Cancer Family Caregiving Experience Model, which conceptualizes caregiving as a dynamic stress process that can affect psychological outcomes [20].

This study found that informal caregivers have a higher unadjusted prevalence of poor mental health and higher odds of poor mental health days than non-caregivers, which echoed other studies’ findings [31–33]. Other research studies contrasting caregivers and non-caregivers [34–36] have similarly identified that caregivers have elevated rates of poor mental health outcomes. Yet, other studies comparing informal caregivers with non-caregivers have found more modest differences or have suggested that caregiving does not necessarily lead to poorer mental health outcomes, although these studies often used different outcome measures, such as life satisfaction [37–39]. Heterogeneity in caregiving experiences also likely explains these differences. For instance, informal caregivers caring for patients with aggressive cancers such as pancreatic cancer often report worse psychological outcomes compared to caregivers caring for patients with other types of cancer, such as breast [40–42]. Additionally, a recent analysis of BRFSS data from 2015–2016 to 2021–2022 identified an increase in FMD among both informal caregivers and the population as a whole [43], a broader trend that may partially explain some differences in our findings [44]. Nonetheless, the persistence of disparities after adjusting for covariates suggests that informal caregiving contributes to psychological strain. These mixed findings also suggest that future caregiver studies should account for differences in patient acuity and prognosis.

Similarly, informal cancer caregivers in this study had higher odds than non-caregivers of reporting a lifetime depression diagnosis, consistent with prior literature demonstrating elevated rates of depressive disorders among cancer caregivers [45, 46]. However, findings across studies have not been consistent, with some demonstrating smaller differences between caregivers and non-caregivers or no clear association between caregiving and depression diagnosis [37, 39]. This variability in findings reflects differences in measurement (e.g., lifetime diagnosis versus current depressive symptoms) or in access to mental healthcare across samples. Lifetime depression captures a historical clinical diagnosis rather than current distress, and it may be less sensitive to the evolving stressors of caregiving. These findings highlight the need to account for patient acuity, prognosis, caregiver burden, and caregiving intensity in future studies.

### Implications

Altogether, these results indicated that informal caregivers require interventions to improve their physical and psychological health. Such interventions should include bolstering components of the stress process of informal caregivers, including addressing primary stressors such as care demands and patient factors, an appraisal of needs, and cognitive-behavioral responses such as coping and self-care to lower the level of burden and distress, and addressing secondary stressors such as schedule, financial challenges, and fatigue. The findings here echoed those of other studies showing lower mental and physical well-being in informal cancer caregivers, especially at the end of a cancer patient’s life [9, 10]. Oncology and related support teams (palliative, hospice, etc.) should carefully consider their programs and support for informal caregivers, providing anticipatory guidance [2, 9] and paying attention to their mental health needs.

### Limitations

Despite the strengths of this analysis, there are limitations to this analysis, and mental health outcomes are challenging to predict. BRFSS data utilize self-reported information, which introduces recall bias and bias due to social desirability, and the measures of mental distress are not caregiving-specific. Additionally, BRFSS data are cross-sectional, so causal inferences cannot be made. The effect sizes (Cramér’s *V*) were weak to moderate in most of the weighted unadjusted analyses, suggesting minor associations. Further, not all states reported on the caregiving modules, and some states had low response rates, which limits generalizability. The caregiving modules also did not assess cancer characteristics such as type or stage. The BRFSS depression question assesses any lifetime depressive diagnosis and does not distinguish between minor and major depressive conditions. Also, comorbid conditions of individuals receiving informal caregiving support could not be assessed in the single-item measure of the BRFSS, nor could the mental and physical health of the informal caregiver before becoming a caregiver.

## Conclusion

This study fills an important gap in the literature with data supporting that poor mental health outcomes are pervasive among U.S. informal cancer caregivers, identifying associated factors with mental health outcomes. The results of this study can inform the public health approach to health promotion by determining further research and interventions to identify relevant issues and ultimately address informal cancer caregivers’ mental health. Future studies should assess caregiving intensity, cancer type, trajectories of stress over time, the interaction of multiple patient comorbidities, in order to develop tailored interventions.

## Supplementary Information

Below is the link to the electronic supplementary material.

Supplementary file1 (DOCX 19 KB)

## Data Availability

Data used are publicly available at https://www.cdc.gov/brfss/index.html
