# Supplementary material for: Poor mental health days and depressive disorders comparing non-caregivers, informal non-cancer caregivers, and informal caregivers of cancer patients: BRFSS 2022–23
Source: Support Care Cancer. 2026 Oct 14;34(11):1100. doi: 10.1007/s00520-026-11337-y (PMC13669027; doi:10.1007/s00520-026-11337-y)
Supplement: Supplementary file 1 [file 520_2026_11337_MOESM1_ESM.docx]

Supplementary Table 1. States included by year, population receiving BRFSS caregiver module, and total cancer caregivers by state for 2022-23

| **State** | **Total population receiving questionnaire versions with caregiver module** | **2022-23 total number of cancer ICs** |
| --- | --- | --- |
| Arizona | 16,902 | 268 |
| Arkansas | 3,666 | 71 |
| Colorado | 3,908 | 51 |
| Georgia | 7,159 | 133 |
| Hawaii | 7,192 | 109 |
| Idaho | 6,332 | 98 |
| Iowa | 4,056 | 49 |
| Louisiana | 9,405 | 208 |
| Maine | 5,430 | 111 |
| Maryland | 4,562 | 69 |
| Mississippi | 3,772 | 67 |
| New Hampshire | 5,706 | 82 |
| New York | 5,041 | 75 |
| Ohio | 33,004 | 500 |
| Oklahoma | 2,488 | 30 |
| Oregon | 10,150 | 131 |
| Pennsylvania | 3,651 | 50 |
| Puerto Rico | 5,357 | 51 |
| Tennessee | 4,781 | 88 |
| Texas | 8,050 | 123 |
| Utah | 8,444 | 122 |
| Virginia | 8,361 | 127 |
| Washington | 22,968 | 312 |
| Wisconsin | 9,279 | 153 |
| **Total** | **199,664** | **3,078** |
